# The Genetic Basis for the Increased Prevalence of Metabolic Syndrome among Post-Traumatic Stress Disorder Patients

**DOI:** 10.3390/ijms232012504

**Published:** 2022-10-19

**Authors:** Burook Misganaw, Ruoting Yang, Aarti Gautam, Seid Muhie, Synthia H. Mellon, Owen M. Wolkowitz, Kerry J. Ressler, Francis J. Doyle, Charles R. Marmar, Marti Jett, Rasha Hammamieh

**Affiliations:** 1 Medical Readiness Systems Biology, Center for Military Psychiatry and Neuroscience, Walter Reed Army Institute of Research, Silver Spring, MD 20910, USA; 2 Vysnova Partners, Inc., Landover, MD 20785, USA; 3 The Geneva Foundation, Silver Spring, MD 20910, USA; 4 Department of Obstetrics, Gynecology & Reproductive Sciences, University of California, San Francisco, CA 94143, USA; 5 Department of Psychiatry and Behavioral Sciences, University of California, San Francisco, CA 94143, USA; 6 McLean Hospital, Belmont, MA 02478, USA; 7 Department of Psychiatry, Harvard Medical School, Boston, MA 02115, USA; 8 Harvard John A. Paulson School of Engineering and Applied Sciences, Harvard University, Cambridge, MA 02134, USA; 9 Department of Psychiatry, New York University Grossman School of Medicine, New York, NY 10016, USA

**Keywords:** PTSD (post-traumatic stress disorder), metabolic syndrome, MDD (major depressive disorder), genetic correlation, obesity

## Abstract

Post-traumatic stress disorder (PTSD) is a highly debilitating psychiatric disorder that can be triggered by exposure to extreme trauma. Even if PTSD is primarily a psychiatric condition, it is also characterized by adverse somatic comorbidities. One illness commonly co-occurring with PTSD is Metabolic syndrome (MetS), which is defined by a set of health risk/resilience factors including obesity, elevated blood pressure, lower high-density lipoprotein cholesterol, higher low-density lipoprotein cholesterol, higher triglycerides, higher fasting blood glucose and insulin resistance. Here, phenotypic association between PTSD and components of MetS are tested on a military veteran cohort comprising chronic PTSD presentation (*n* = 310, 47% cases, 83% male). Consistent with previous observations, we found significant phenotypic correlation between the various components of MetS and PTSD severity scores. To examine if this observed symptom correlations stem from a shared genetic background, we conducted genetic correlation analysis using summary statistics data from large-scale genetic studies. Our results show robust positive genetic correlation between PTSD and MetS (r_g_[SE] = 0.33 [0.056], *p* = 4.74E-09), and obesity-related components of MetS (r_g_ = 0.25, SE = 0.05, *p* = 6.4E-08). Prioritizing genomic regions with larger local genetic correlation implicate three significant loci. Overall, these findings show significant genetic overlap between PTSD and MetS, which may in part account for the markedly increased occurrence of MetS among PTSD patients.

## 1. Introduction

Post-traumatic stress disorder (PTSD) is a highly debilitating psychiatric disorder that can develop after experiencing extreme traumatic event [1,2]. Besides trauma exposure, PTSD is defined and diagnosed with four additional criteria of symptom clusters (re-experiencing, hyperarousal, negative alterations in cognitions and mood, and avoidance) [3]. It occurs at an estimated rate of 6.8–8% among the general public in the US [4,5] and at a rate of 10–30% among military members [6,7]. Its incidence rate is also particularly higher among female and individuals with adverse childhood experience [1].

While PTSD is classified as a mental disorder, chronic PTSD is characterized by somatic comorbidities that affect the whole-body system besides the brain [8,9]. One of the most commonly co-occurring condition with PTSD is metabolic syndrome (MetS). MetS refers to a constellation of abnormalities related to metabolic dysfunction [10]. Its components include obesity (particularly abdominal), dyslipidemia (particularly lower HDL-C [high-density lipoprotein cholesterol], higher LDL-C [low-density lipoprotein cholesterol], higher triglycerides), elevated blood pressure and hyperglycemia including higher fasting blood glucose [FBG] and insulin resistance. Observational studies using epidemiological data showed a clear association between PTSD and metabolic syndrome as a whole and its components individually [9,11,12].

Besides the higher co-occurrence with PTSD, another intriguing fact is the significant phenotypic and genetic association of MetS with other stress-related psychiatric disorders including major depressive disorder (MDD). MDD is a mood disorder that shares a lot of symptomatology with PTSD. In addition to the substantial symptom overlaps, PTSD and MDD also have high genetic overlap [13,14]. Various aspects of the association between MDD and MetS have been extensively studied, showing a clear epidemiological co-occurrence as well as significant genetic overlap [15,16]. Some Mendelian randomization studies also suggested the direction of influence is from MDD to MetS [17]. However, it is not clear whether the genetic relationship between PTSD and MetS resembles that of between MDD and MetS.

Most studies pertaining to understanding the relationship between PTSD and MetS are limited to showing their epidemiologic co-occurrence. Fundamental questions as to the nature of this commonly observed comorbidity are yet to be addressed. What is the relationship between PTSD severity and level of metabolic dysregulation? How much of observed association between PTSD phenotypes and MetS as a whole and its components stem from genetic underpinning? What is the direction of causality, if there is any, between these associations? Owing to the high prevalence and the detrimental consequences of these disorders, answers to these questions have important practical implications. These include how to judiciously prioritize treatments targeting a particular illness from among a set of comorbidities so as to maximize benefit in terms of the overall health of an individual. For example, in the absence of causal influences between co-occurring disorders, targeting one condition will not have a direct impact on reducing risk for the other. 

Genetic studies of the psychiatric and metabolic traits have recently witnessed analysis with large-scale sample sizes [18,19,20,21]. The resulting summary statistics, which are often publicly available, can account for a significant proportion of the heritability of these complex polygenic traits, enabling various kinds of interrogation of the genetic aspect of disease mechanisms. The current study tries to answer the aforementioned research questions using large-scale publicly available GWAS (genome-wide association study) summary statistics data as well as data derived from our own studies.

In this study, we aim to enhance our understanding of the link between PTSD and MetS as well as its components. First, we test the phenotypic association between PTSD phenotypes (PTSD status and symptom severity scores) and multiple metabolic traits using data from the PTSD Systems Biology Consortium (SBC) cohort, a well-characterized cohort comprising US veterans of the Iraq and Afghanistan wars. Then, we estimate genetic correlations between PTSD and metabolic traits using data from large-scale GWAS summary statistics. We also identified pleiotropic genetic regions contributing to the shared genetics between PTSD and MetS. Finally, in order to gain insight into the presence and direction of causal influences between Mets and psychiatric disorders, we conduct Mendelian randomization analysis. 

## 2. Results

### 2.1. Association between PTSD Phenotypes and Components of Metabolic Syndrome

**Figure 1:**
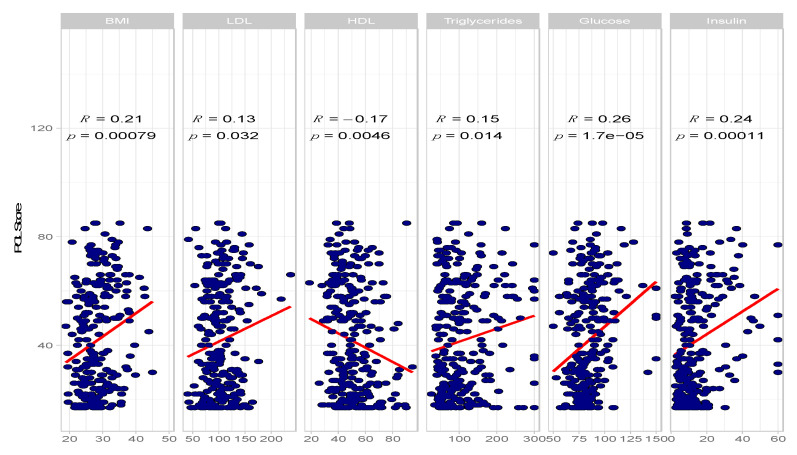
Phenotypic associations between MetS components and PCL score.

The co-occurrence between PTSD and MetS has been reported in multiple studies including in meta-analysis [9,22]. Besides MetS diagnosis, all of its components have also been shown to be associated with PTSD individually. We tested the magnitude of correlations among components of MetS and PTSD severity scores on 310 participants from the SBC cohort comprising military veterans with chronic PTSD presentation. We tested both self-reported PTSD Checklist (PCL) as well as clinician-administered PTSD (CAPS) PTSD severity scales. Six variables related to MetS were measured for the SBC participants: BMI, LDL-C, HDL-C, triglycerides, fasting glucose and insulin. As expected, all of the measured MetS-related measurements are significantly correlated with PTSD severity scores (Figure 1 and Table 1). The strongest associations are for fasting glucose with total PTSD symptom scores on the PCL (r = 0.26, *p* = 1.7E-05 and CAPS (r = 0.32, *p* = 9E-08). 

**Figure 2:**
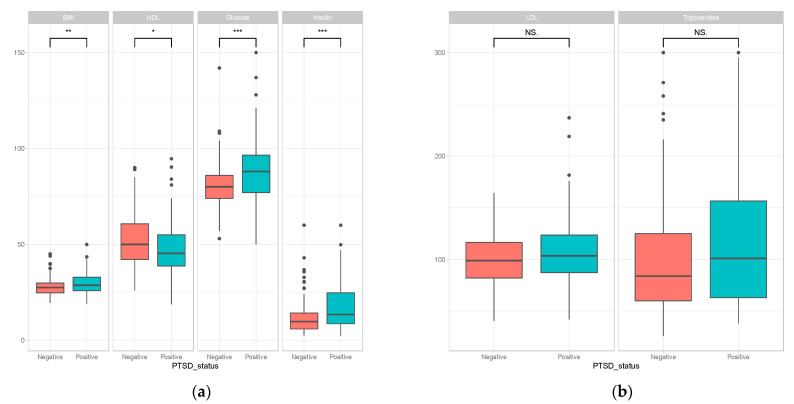
Comparing levels of components of MetS between PTSD cases and healthy controls. (**a**) Some MetS components show statistical difference between PTSD cases and healthy controls. (**b**) Some MetS components show change in the expected direction between PTSD-positive cases and PTSD-negative controls but not at statistically significant level. (***: *p* < 0.0001, **: *p* < 0.01, *: *p* < 0.05, NS: *p* > 0.05).

Owing to the inclusion criteria of the study recruitment, the distribution of the PTSD symptom severity scores seems to have a bimodal distribution (consists of values at the two extreme values but not in the middle). To probe the effect of this distribution on the observed association, we performed additional tests using the non-parametric Spearman rank correlation test. Results from Spearman rank correlation test also showed significant association between PTSD severity scores and the various components of MetS (Table 1). Analysis with binary PTSD diagnosis label, instead of a quantitative PTSD severity score, resulted in a significant association for most of the MetS relevant variables (Figure 2).

In order to see the effect of chronicity of the PTSD on its relationship with metabolic dysregulation, we sought to examine these associations in a cohort comprising PTSD symptoms from more recent trauma exposure. Unlike the clear association between metabolic dysregulation with long-term PTSD presentation, using a cohort comprising more recent onset PTSD diagnosis from recent trauma-exposures (FCC; *n* = 1134), we did not find a similar pattern of association between the same MetS components and PCL score (Figure S1). These observations are consistent with the hypothesis that PTSD triggers MetS susceptibility, rather than the other way around. That is, if the direction of influence were from MetS components to PTSD, we would expect to see significant association in both chronic and short-duration PTSD presentations. Besides the difference in duration of PTSD, the FCC participants are active-duty service members that are much younger (7.6 years younger on average) than SBC participants (Table S1).

### 2.2. Genome-Wide Genetic Correlations between PTSD and Metabolic Traits

In order to determine whether these phenotypic associations stem from a shared genetic underpinning, we estimated pairwise genetic correlations between PTSD and MetS as well as the individual components of MetS. Based on GWAS summary statistics from recent large-scale studies on MetS-related traits with European-ancestry participants, SNP-array based heritability estimates were computed to ensure they are sufficiently powered and large enough proportion of the phenotype variance is explained. All summary statistics used for further analysis have SNP heritability estimates of more than 5%. Using estimates from LD score regression (LDSC), there is evidence for a statistically significant genetic correlation between PTSD diagnosis and MetS (r_g_[SE] = 0.3305 [0.0564], *p* = 4.74E-09). 

Obesity-related components (BMI and WHR) also show statistically significant genetic correlations. On the other hand, the remaining components of MetS in which we investigated genetic correlation estimates did not pass Bonferroni multiple-testing corrected *p*-value significance threshold (*p* < 0.05/8 = 0.00625). Despite the strong phenotypic correlation between PTSD and sugar-related traits, the current study did not find corresponding statistically significant genetic correlation. This can be due to the fact that the GWAS summary statistics for these two traits are much less powered (about half the SNP heritability) compared to the other traits. Details of genetic correlation estimates between PTSD and various metabolic traits are listed in Table 2.

**Figure 3:**
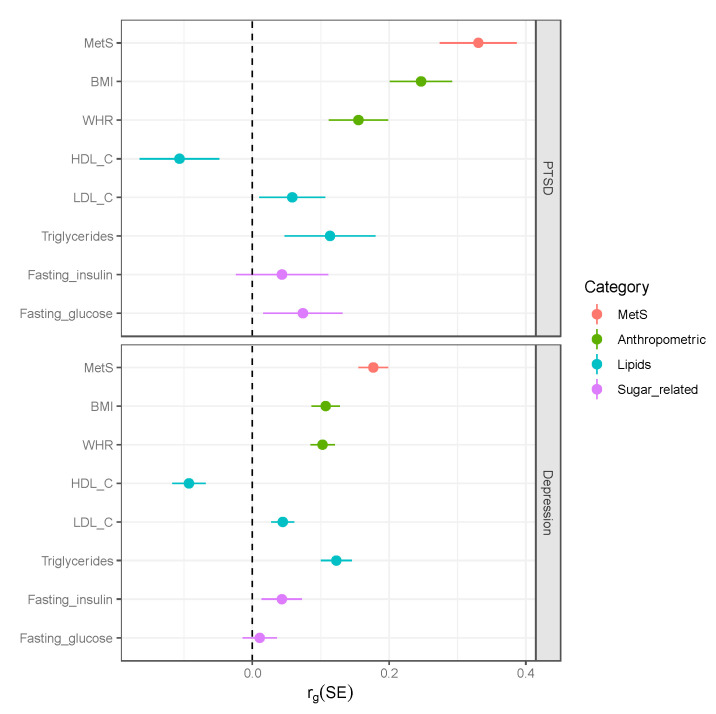
Genetic correlation estimates between MetS and its components with PTSD (top) and depression (bottom). The bars correspond to standard errors (SE) and the genetic correlation estimates (r_g_) are represented by a dot in the middle of the SE bars.

Some previous studies reported significant genetic correlation between MetS and depression phenotypes [17,23]. To compare estimates of genetic associations of MetS and PTSD with genetic associations of MetS and MDD, we computed genetic correlation estimates with MDD using the same summary statistics of metabolic traits. Our analysis confirmed previously reported genetic correlations between MDD and metabolic traits. We observed genetic correlation estimates with PTSD are comparable but slightly stronger than estimates for MDD (Figure 3).

### 2.3. Genomic Regions with Significant Local Genetic Correlation between PTSD and MetS

**Figure 4:**
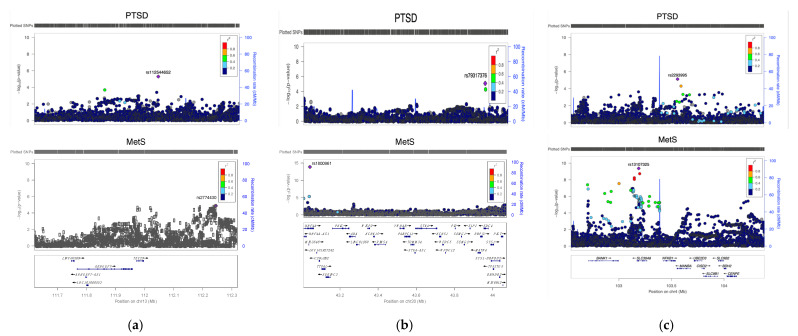
Regional association plots for three loci with significant local genetic correlation between PTSD and MetS. (**a**) 13:111,621,245–112,319,064, (**b**) 20:43,008,891–44,072,210, (**c**) 4:102,544,804–104,384,534.

Given the significant genome-wide genetic correlation between PTSD and MetS, we sought to identify specific genomic regions and genes with significant contribution to the globally aggregated genetic correlation. Three genomic regions with significant local genetic correlation are identified: (i) chromosome 13:111.62–112.32 Mb (r_g,local_ = 0.707, *p*= 3.30 E-05), (ii) chromosome 20:43.01–44.07 Mb (r_g,local_ = 0.754, *p* = 2.99E-04), and (iii) chromosome 4:102.54–104.38 Mb (r_g,local_ = 0.609, *p* = 7.99E-04). These regions contain genes previously implicated in genetic studies of relevant traits including SLC39A8 and HNF4A (Figure 4). Notably, one of the identified loci (4:102.54–104.38 Mb) has previously been shown to be one of the regions underlying shared genetic liability between psychiatric and immune-related phenotypes [24].

### 2.4. Bidirectional Two-Sample Mendelian Randomization Analysis

Since the metabolic traits are correlated with chronic but not short-duration PTSD, we hypothesized the direction of causal influence might be from PTSD to metabolic dysregulation, but not the reverse. To test this hypothesis, we first selected 16 genome-wide significant (*p* < 1E-08) and LD independent (pruning with r^2^ threshold of 0.1 and window of 10 Mb) SNP’s as genetic instruments. Testing the causal hypothesis that PTSD results in MetS using the inverse variance weighted (IVW) Mendelian randomization method did not result in a statistically significant estimate (b = 0.095, SE = 0.176, *p* = 0.59). Similarly, using 146 genome-wide significant and independent SNP’s in the MetS summary statistics, a test of the effect of MetS on PTSD also did not pass a significant threshold (b = 0.017, SE = 0.009, *p* = 0.06).

Next, since we could not determine the direction of influence between PTSD and MetS despite the significant genetic correlation, we sought to assess the direction of influence with MDD, which is another stress-related psychiatric disorder with large-scale well-powered genetic study for Mendelian randomization analysis. A similar IVW analysis suggest the direction of influence is from MDD to MetS (40 genetic instruments, b = 0.23, SE = 0.06, *p* = 1.3E-04) rather than from MetS to MDD (108 genetic instruments, b = 0.015, SE = 0.011, *p* = 0.165). This comports with previous reports of the effect of depression phenotypes on metabolic dysregulation [17,23].

## 3. Discussion

PTSD often co-occurs with other psychiatric disorders (including depression and anxiety disorders) and physical ailments (including metabolic syndrome, cardiovascular diseases, and type-2 diabetes) [9,25]. Understanding the relationship among these comorbidities is crucial for effectively reducing their detrimental impact. In the present study, we investigated the phenotypic association and genetic overlap between PTSD and one of its common comorbidities, MetS. First, we showed that level of metabolic dysfunction increases with severity of PTSD symptoms. We found genetic correlation estimates closely reflect phenotypic associations (in line with Cheverud’s conjecture [26]) even if they are estimated using completely independent datasets. Moreover, the phenotypic association between MetS and long-term PTSD presentation but not with the shorter duration PTSD presentation is consistent with the Mendelian randomization finding that psychiatric illnesses play a causal role in increasing MetS onset, but not necessarily the reverse. This direction of causality suggest effective PTSD/MDD treatment may help mitigate the onset of MetS-related risk factors.

Some clinical implications of the results of the present study are worth mentioning. First, it is important to acknowledge that the relationship between PTSD and MetS is not limited to increased preponderance in binary case–control comparison. Higher PTSD symptom severity scores are also associated with elevated level of metabolic dysfunction. Second, the epidemiologic link between PTSD and metabolic syndrome may arise from a shared genetic underpinning. Future risk stratification and management strategies can benefit from incorporating this insight. Third, the level of genetic overlap between metabolic traits with PTSD is comparable to that of major depression. Fourth, the direction of causal influence is likely from the psychiatric illnesses leading to metabolic dysfunction, but may not be the other way around. Therefore, interventions that target the psychiatric disorders may also help reduce metabolic-related disease risk, which are some of the leading causes of mortality in the general public. In fact, the high comorbidity with MetS and its components is thought to be a major factor responsible for the markedly high (2 to 3 times higher) mortality rate among PTSD patients [9,27]. In order to design effective intervention strategies, the etiologic structure underlying these commonly co-occurring conditions need to be deciphered. The sequence of pathological events among these comorbidities, for example, will inform which illness should be targeted so as to maximize benefit for the other comorbidities as well and overall health condition. 

MetS is a major risk factor for adverse cardiovascular events, diabetes and all-cause mortality [28,29]. Its individual components are also independently associated with cardiovascular diseases and the occurrence of multiple concurrent MetS risk factors are shown to increase the severity of the associated cardiovascular disease [30]. For instance, risk for stroke and coronary heart disease increases threefold, while cardiovascular mortality increases more than fivefold among those with MetS compared to those without [31]. Therefore, a better understanding of the nature of the relationship between PTSD and MetS may yield insight into the mechanism of the markedly elevated mortality rate among PTSD patients. In particular, the findings in the present study suggest metabolic dysfunction may play a mediating role for the significant association between PTSD and cardiovascular diseases [32].

Besides the overall MetS diagnosis, the MetS-related metabolic traits have previously been reported to be associated with PTSD phenotypes. PTSD is reported to be significantly associated with higher BMI, and particularly with central obesity [33,34]. Multiple studies including meta-analysis showed PTSD patients have higher levels of LDL-C and triglycerides, but lower level of HDL-C compared to healthy controls [35]. Increased fasting blood glucose (hyperglycemia) and insulin are also observed among PTSD patients [12], [36]. There are also strong evidence depicting strong association between these metabolic traits and major depression, which is a common comorbidity of PTSD [37,38]. However, some studies suggest these associations may not be uniform across gender and age groups and sub-population specific patterns need to be deciphered [39].

This study involved estimating the level of genome-wide correlations. Future studies that can identify specific polymorphisms, genes, pathways and metabolites involved in the common pathophysiology of PTSD and metabolic dysfunction are needed. In particular, abundance of many metabolites (a molecular phenotype that is closest to physiological and clinical endpoints among the cascade of biological processes) is shown to be highly genetically influenced [40,41], making it amenable to the kind of approaches we conducted in this study. Blood concentration levels of many small molecule biochemical (including metabolites) correlates of PTSD/MDD [11,42,43] have high heritability estimates, both from twin/family studies [44] as well as SNP array-based studies [41]. Some preclinical animal studies and observational studies with human data pointed to the association of many metabolite levels to PTSD and its psychiatric comorbidities.

The findings of the present study should be interpreted in light of its strengths and limitations. Most previous studies focused on comparing the presence and prevalence of MetS and associated metabolic traits between PTSD patients with healthy controls. Here, the phenotypic associations with PTSD were assessed using two quantitative scores of PTSD symptom severity. This approach can capture the effect of disease severity in addition to a binary disease onset. Another strength of the study is that the genetic correlation estimates are obtained from large-scale genetic analysis results. To ensure this summary statistics are well-powered, SNP heritability of the summary statistics data are computed and only those with sufficient minimum threshold are used, resulting in a more reliable genetic correlation estimate. 

The main limitations of the study are the following. First, to avoid population stratification effects, only genetic studies with European-ancestry participants are collected and analyzed. Therefore, the results with this summary data may not necessarily generalize on other ancestral groups. Second, genetic correlations between psychiatric conditions and metabolic traits are reported to be gender and age dependent [45]. In the present study, we could not tease out sub-population specific signals using our current data. The findings in the present study should be interpreted in light of these and other potential confounding variables that could not be controlled. Third, the Mendelian randomization results should be seen as an exploratory analysis that need to be thoroughly reanalyzed in future studies with appropriately sufficient sample sizes. 

PTSD, MetS and its components are heritable traits with large-scale publicly available GWAS summary statistics data, enabling application of modern genetic analysis tools to interrogate the mechanism of influence among them [13,46]. In this study, we conducted four sets of distinct but interrelated analysis. First, we confirmed phenotypic association between PTSD phenotypes and components of MetS on a well-characterized military veteran cohort comprising chronic PTSD presentations. Then, using publicly available GWAS summary statistics data, we showed significant genetic correlations between PTSD diagnosis and MetS as a whole and some of its components. We also conducted local genetic correlation analysis to prioritize genetic regions with significant pleiotropic effect on PTSD and MetS. Finally, we implemented a bidirectional two-sample Mendelian randomization analysis to test the direction of causal influence between MDD and MetS. Our analysis suggests metabolic dysfunction is a detrimental sequalae of stress-induced psychiatric illnesses, and may be one of the mechanisms leading to the high mortality rate among MDD and PTSD patients. 

Future studies need to further investigate many aspects of the pathophysiology of the co-occurrence of PTSD and MetS. These include (i) replicating and refining the current findings with additional datasets as results from new larger cross-ancestry genetic studies become available, (ii) identifying metabolites and other small molecule biochemicals that mediate the effect of genetics on PTSD/MDD, (iii) uncovering specific genes and pathways underpinning the genetic correlations between PTSD/MDD and metabolic dysregulation, and (iv) identifying potential pleiotropic genetic variants that are shared between PTSD and metabolite genetics.

## 4. Materials and Methods

**Figure 5:**
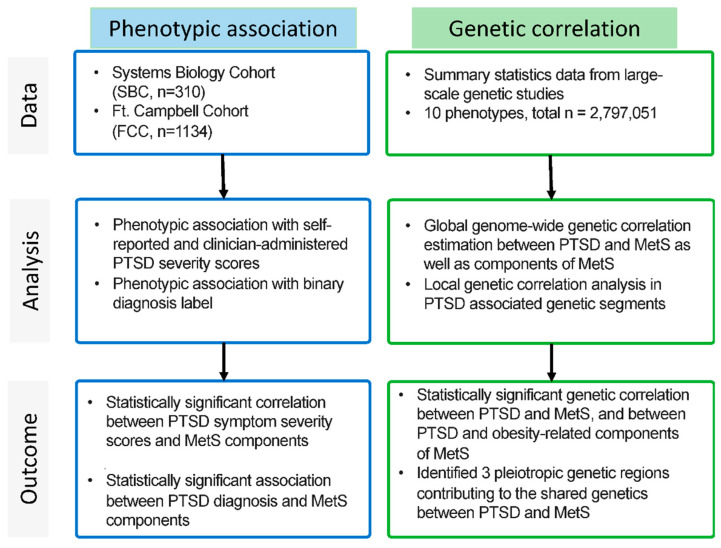
Workflow of the study.

The current study involves examining the relationship between PTSD and metabolic dysregulation at genetic and phenotypic levels. These two aspects are done in parallel on independent datasets (Figure 5).

### 4.1. Systems Biology Consortuim (SBC) and Fort Campbell Cohort (FCC) Datasets

The SBC (Systems Biology Cohort) consists of participants from US military veterans who served in OEF (Operation Enduring Freedom) and/or OIF (Operation Iraqi Freedom) [47,48]. All participants in the PTSD-positive cases and PTSD-negative controls experienced DSM-IV PTSD Criterion A trauma (combat exposure). Two PTSD assessment criteria were implemented for SBC participants. The first one, CAPS (Clinician Administered PTSD Scale) for DSM-IV, is a structured questionnaire administered by a clinician to assess PTSD symptom severity [49]. The second one, PCL (PTSD Checklist), is a self-administered 17-item questionnaire to estimate PTSD severity and ascertain PTSD diagnosis [50]. Exclusion criteria include suicidal ideation, substance abuse within the past year, history of alcohol dependence within the past eight month of PTSD assessment, a lifetime history of any psychiatric disorder with psychotic features, bipolar disorder, or obsessive compulsive disorder, any neurological disorder, systemic illness affecting central nervous system function, and history of a moderate or severe traumatic brain injury. Multiple metabolic traits are measured for all participants: body-mass index (BMI), low-density lipoprotein cholesterol (LDL-C), high-density lipoprotein cholesterol (HDL-C), triglycerides, fasting glucose and insulin. More details of the SBC cohort are shown in Table 3. 

The FCC (Fort Campbell Cohort) participants comprises active-duty service members in the US Army with recent PTSD symptom presentation from recent trauma exposure (within 90–180 days of return from deployment) [51]. Given the larger sample size (*n* = 1134) of the cohort, only PCL is available to assess PTSD symptoms. Metabolic traits (BMI, LDL-C, HDL-C, triglycerides and insulin) are also measured in the same manner as the SBC dataset. Written informed consent was obtained from all participants in both cohorts and approvals were obtained from all appropriate Institutional Review Boards.

### 4.2. GWAS Summary Statistics Data

Several large-scale GWAS summary statistics pertaining to MetS or its components as well as PTSD and depression are obtained from public repositories. The PTSD GWAS summary statistics is from the Psychiatric Genomic Consortium (PGC) freeze-2 study of the European-ancestry participants [18]. The study consists of 23,212 PTSD-positive cases and 151,447 healthy controls and provided univariate association test results for 9.77 M genetic variants. The depression summary statistics [52] consists of combined samples from PGC-MDD [20] and UKBB depression [53] studies. Details of the summary statistics that are used in the current study are summarized in Table 4.

Preprocessing and quality-control step is done on each of the summary statistics individually as follows. Rare variants (minor allele frequency, MAF < 0.01) are filtered out. If z-statistics is not given, it is computed from the effect size (regression coefficient or odds-ratios) of the univariate tests (Z = beta/SE or Z = log(OR)/SE). If rsID’s of genetic variants are not provided in the original summary statistics, its rsID is looked up from dbSNP database and inserted as an additional column. After computing SNP array heritability on the given summary statistics, if it does not explain a significant variance of the phenotype ($h_{SNP}^{2}<5\%$), that summary statistics is dropped from further analysis. 

### 4.3. Estimating Genome-Wide Genetic Correlations and SNP Heritability

The proportion of variance of the association between two genetically influenced traits that is attributable to genetic background is known as genetic correlation (r_g_). Its estimate is obtained from GWAS summary statistics data using cross-trait LD score regression (LDSC) [59]. A Python command line implementation of LDSC was used. Genome-wide SNP array heritability (the fraction of phenotypic variance explained by genetic variation) of the summary statistics are also computed using LDSC. LD scores that was precomputed for European ancestry individuals in the 1000 Genome Project was used [60].

### 4.4. Estimating Local Genetic Correlations on Independent LD Blocks

A predefined LD blocks that was defined using European European-ancestry 1000-genome data is obtained [61]. The whole human genome is divided into 2495 LD independent segments in such a way that each block of ~1 Mb are independent of each other. Genomic regions containing at least one SNP that is suggestively significant (*p* < 1E-05) associations with PTSD are selected, resulting in 50 independent regions for further analysis. Univariate local heritability analysis on these 50 genomic regions was conducted for PTSD susceptibility. A total of 43 regions with Bonferroni corrected significance (*p* < 0.05/50 = 0.001) are selected for further bivariate local genetic correlations. Local genetic correlation estimates between PTSD and MetS are analyzed on these 43 genomic regions. Both univariate local heritability and bivariate local genetic correlation analysis are conducted with LAVA (Local Analysis of coVariant Association) [61]. Regional association plots are generated with LocusZoom [62]. All genomic locations are based on coordinates in the hg19/GRCh37 human genome reference. 

### 4.5. Mendelian Randomization Analysis

Genetic instrumental variables are selected based on association statistics on the exposure phenotype summary statistics (PTSD [18], MetS [21], and depression [20]). Only genome-wide significant SNP’s (*p* < 5E-08) are selected. To enforce independence among genetic instruments, LD clumping procedure with window distance of 10 Mb and r^2^ threshold of 0.1 is conducted. For each instrumental variable, the corresponding SNP-outcome association statistics is obtained from the three summary statistics. After ensuring the same effect allele is used in the exposure and outcome summary statistics, palindromic SNP pairs (C/G and A/T alleles) are removed.

Two-sample (separate sources of GWAS summary statistics for the exposure and outcome variables) Mendelian randomization analysis is performed using the inverse variance weighted (IVW) method [63]. Briefly, IVW aggregates the overall effect by summing the weighted (where the weights are the reciprocal of the variances) estimate from individual genetic instrument (which is the Wald ratio of the exposure to outcome effect). The Mendelian randomization analysis is done with TwoSampleMR package [64] in R (version 4.1.1) statistical software. 

## Figures and Tables

**Table 1:** Correlation estimates between CAPS score and metabolic traits in the SBC dataset.

		Pearson Correlation		Spearman Rank Correlation	
var1	var2	r	*p*	r	*p*
CAPS	BMI	0.22	3.58E-04	0.26	1.45E-06
CAPS	LDL-C	0.14	1.82E-02	0.14	2.08E-02
CAPS	HDL-C	−0.17	4.42E-03	−0.17	3.92E-03
CAPS	Triglycerides	0.12	5.30E-02	0.14	2.10E-02
CAPS	Glucose	0.25	2.15E-05	0.37	3.34E-10
CAPS	Insulin	0.29	1.10E-06	0.35	2.63E-09

**Table 2:** Genetic correlation estimates between PTSD and metabolic traits.

Phenotype	r_g_	SE	*p*
MetS	0.3305	0.0564	4.74E-09
BMI	0.2467	0.0456	6.42E-08
WHR	0.1551	0.0434	3.56E-04
HDL-C	−0.1067	0.0582	6.66E-02
LDL-C	0.0583	0.0482	2.26E-01
Triglycerides	0.1135	0.0667	8.90E-02
Insulin	0.0432	0.0676	5.23E-01
Glucose	0.0739	0.0581	2.04E-01

**Table 3:** Descriptive table for SBC cohort.

	Negative (n = 164)	Positive (n = 146)	Overall (n = 310)
CAPS total score			
Mean (SD)	4.00 (5.14)	68.1 (18.2)	34.2 (34.6)
Median [Min, Max]	2.00 [0, 19.0]	66.0 [24.0, 114]	15.5 [0, 114]
PCL toral score			
Mean (SD)	25.5 (9.04)	59.6 (12.9)	41.9 (20.3)
Median [Min, Max]	23.0 [17.0, 62.0]	61.0 [25.0, 85.0]	37.0 [17.0, 85.0]
Missing	24 (14.6%)	16 (11.0%)	40 (12.9%)
BDI total score			
Mean (SD)	5.85 (6.46)	24.3 (11.0)	14.9 (12.9)
Median [Min, Max]	3.00 [0, 28.0]	24.0 [0, 56.0]	13.0 [0, 56.0]
Missing	24 (14.6%)	11 (7.5%)	35 (11.3%)
Gender			
Female	29 (17.7%)	25 (17.1%)	54 (17.4%)
Male	135 (82.3%)	121 (82.9%)	256 (82.6%)
Age			
Mean (SD)	33.1 (7.90)	33.5 (7.96)	33.3 (7.91)
Median [Min, Max]	30.0 [20.0, 59.0]	31.0 [23.0, 59.0]	31.0 [20.0, 59.0]
Missing	23 (14.0%)	25 (17.1%)	48 (15.5%)
Ethnicity			
Asian	12 (7.3%)	4 (2.7%)	16 (5.2%)
Black	41 (25.0%)	49 (33.6%)	90 (29.0%)
White	76 (46.3%)	53 (36.3%)	129 (41.6%)
Other	12 (7.3%)	15 (10.3%)	27 (8.7%)
Missing	23 (14.0%)	25 (17.1%)	48 (15.5%)
BMI			
Mean (SD)	27.9 (4.51)	29.5 (5.49)	28.6 (5.07)
Median [Min, Max]	27.5 [19.5, 45.0]	28.6 [18.9, 49.9]	27.9 [18.9, 49.9]
Missing	27 (16.5%)	15 (10.3%)	42 (13.5%)
HDL-C			
Mean (SD)	52.0 (13.8)	48.4 (12.9)	50.3 (13.5)
Median [Min, Max]	50.0 [25.9, 90.0]	45.6 [18.8, 94.6]	48.8 [18.8, 94.6]
Missing	21 (12.8%)	15 (10.3%)	36 (11.6%)
LDL-C			
Mean (SD)	99.8 (25.4)	107 (31.5)	103 (28.6)
Median [Min, Max]	99.0 [40.6, 164]	103 [42.0, 237]	100 [40.6, 237]
Missing	21 (12.8%)	17 (11.6%)	38 (12.3%)
Triglycerides			
Mean (SD)	107 (83.3)	122 (82.6)	114 (83.2)
Median [Min, Max]	84.0 [26.0, 718]	101 [38.0, 492]	92.0 [26.0, 718]
Missing	21 (12.8%)	14 (9.6%)	35 (11.3%)
Glucose			
Mean (SD)	80.5 (11.7)	91.1 (28.4)	85.6 (22.0)
Median [Min, Max]	80.0 [53.0, 142]	88.0 [50.0, 309]	83.0 [50.0, 309]
Missing	21 (12.8%)	14 (9.6%)	35 (11.3%)
Insulin			
Mean (SD)	11.6 (8.84)	18.8 (16.5)	15.1 (13.6)
Median [Min, Max]	9.75 [2.40, 67.4]	13.3 [2.20, 108]	11.1 [2.20, 108]
Missing	22 (13.4%)	14 (9.6%)	36 (11.6%)

**Table 4:** GWAS summary statistics used in the current study.

Category	Sum. Data	Phenotype	Sample Size	$h_{SNP}^{2}$	Source Reference
PSY	PTSD (PGC-freeeze-2)	PTSD	174,659	6.50%	[18]
	Depression (PGC+UKBB)	depression	500,199	6.10%	[52]
MetS	MetS (UKBB)	metabolic syndrome	291,107	9.20%	[21]
Anthropometric	BMI	Body mass index	315,347	16.70%	[54]
	WHR	Waist-to-hip ratio	502,773	13.50%	[55]
Lipids	LDL-C	LDL-C	431,167	18.20%	[56]
	HDL-C (UKBB)	HDL-C	115,082	16.80%	[57]
	Triglycerides	Triglycerides	115,082	19.70%	[57]
Sugar related	Glucose (GIANT)	fasting blood glucose	200,622	8.30%	[58]
	Insulin (MAGIC)	Fasting insulin	151,013	8.10%	[58]

PGC: Psychiatric Genomics Consortium; UKBB: UK Biobank; GIANT: Genetic Investigation of ANthropometric Traits consortium; MAGIC: Meta-Analyses of Glucose and Insulin-related traits Consortium.

## Data Availability

All data for phenotypic association analysis are available with permission through the SysBioCube, at https://sysbiocube-abcc.ncifcrf.gov, (accessed on 1 March 2022). GWAS summary statistics data can be obtained from the source references as cited in the manuscript.

## Supplementary Materials

The following supporting information can be downloaded at: https://www.mdpi.com/article/10.3390/ijms232012504/s1.

## Disclaimer

The views: opinions, and findings contained in this report are those of the authors and should not be construed as official Department of the Army position, policy, or decision, unless so designated by other official documentation. Citations of commercial organizations or trade names in this report do not constitute an official Department of the Army endorsement or approval of the products or services of these organizations. The investigators have adhered to the policies for protection of human subjects as prescribed in AR 70–25.
