# Supplementary material for: The Genetic Basis for the Increased Prevalence of Metabolic Syndrome among Post-Traumatic Stress Disorder Patients
Source: Int J Mol Sci. 2022 Oct 19;23(20):12504. doi: 10.3390/ijms232012504 (PMC9604263; doi:10.3390/ijms232012504)
Supplement: Supplementary file 1 [file ijms-23-12504-s001.zip › ijms-1956300-supplementary.pdf]

International Journal of
Molecular Sciences

MDPI

Supplementary Figures and Tables

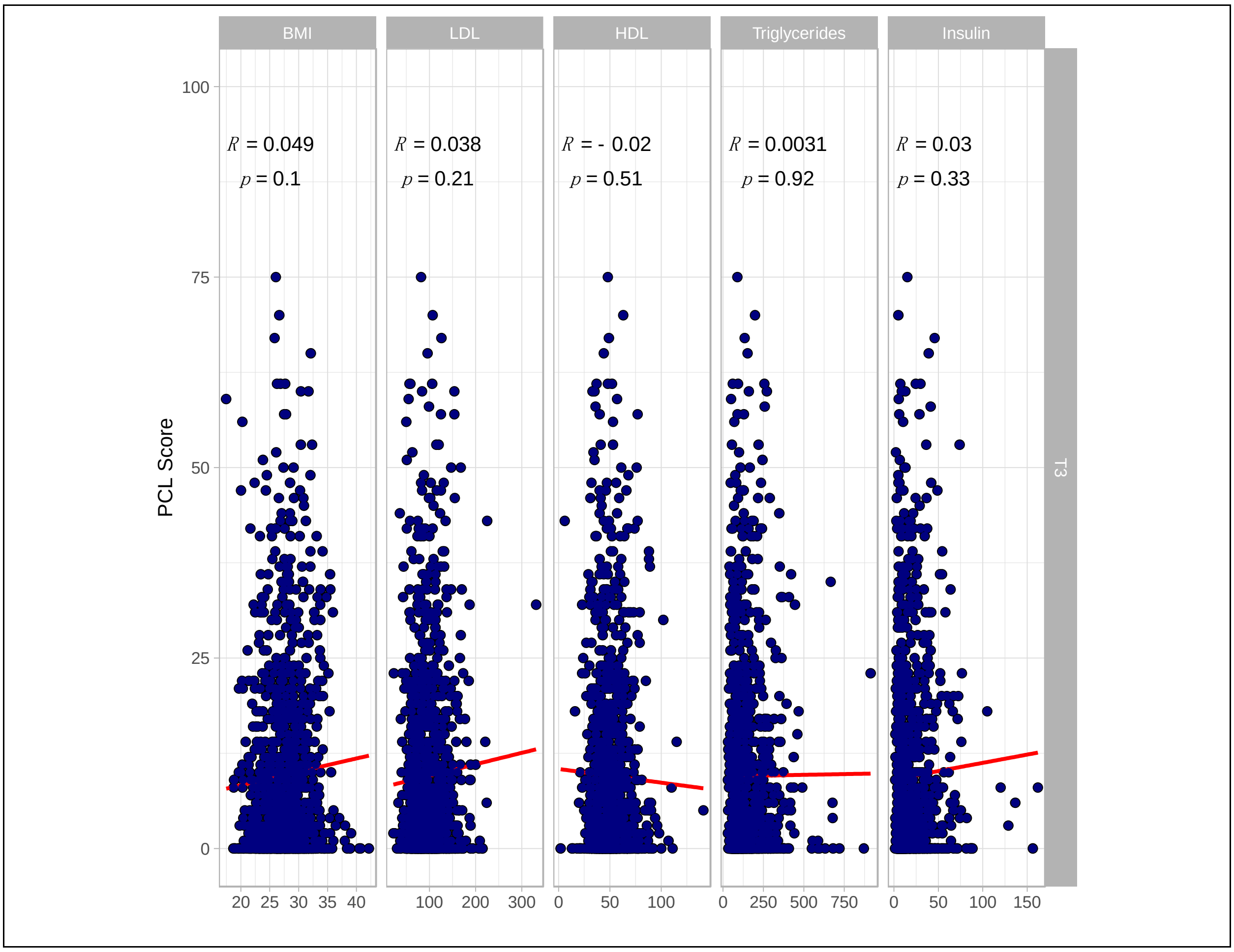

Figure S1. Components of the MetS are not significantly correlated with PTSD severity score in a cohort with recent PTSD symptom presentation.

Table S1. Descriptive table for FCC cohort.

	Negative (N=1030)	Positive (N=104)	Overall (N=1134)
**PCL_score**			
Mean (SD)	6.38 (7.71)	41.7 (10.2)	9.61 (12.9)
Median [Min, Max]	3.00 [0, 30.0]	38.5 [31.0, 75.0]	4.00 [0, 75.0]
**GENDER**			
Female	69 (6.7%)	13 (12.5%)	82 (7.2%)
Male	961 (93.3%)	91 (87.5%)	1052 (92.8%)
**T1_AGE**			
Mean (SD)	25.6 (5.91)	26.5 (6.06)	25.7 (5.92)
Median [Min, Max]	24.0 [18.0, 51.0]	25.0 [20.0, 44.0]	24.0 [18.0, 51.0]
Missing	533 (51.7%)	62 (59.6%)	595 (52.5%)
**RACE**			
Asian	29 (2.8%)	0 (0%)	29 (2.6%)
Black	134 (13.0%)	12 (11.5%)	146 (12.9%)
White	676 (65.6%)	71 (68.3%)	747 (65.9%)
Native American	9 (0.9%)	1 (1.0%)	10 (0.9%)
Hispanic	130 (12.6%)	16 (15.4%)	146 (12.9%)
Other	50 (4.9%)	4 (3.8%)	54 (4.8%)
Missing	2 (0.2%)	0 (0%)	2 (0.2%)
**BMI**			
Mean (SD)	27.4 (3.58)	27.9 (3.55)	27.4 (3.58)
Median [Min, Max]	27.2 [18.7, 42.1]	27.8 [17.5, 35.9]	27.3 [17.5, 42.1]
Missing	12 (1.2%)	1 (1.0%)	13 (1.1%)
**HDL**			
Mean (SD)	50.6 (14.2)	50.0 (15.0)	50.5 (14.3)
Median [Min, Max]	49.0 [2.00, 141]	48.0 [6.00, 89.0]	49.0 [2.00, 141]
Missing	17 (1.7%)	3 (2.9%)	20 (1.8%)
**LDL**			
Mean (SD)	97.9 (31.9)	102 (40.7)	98.3 (32.8)

Table S1. Descriptive table for FCC cohort.

	Negative (N=1030)	Positive (N=104)	Overall (N=1134)
Median [Min, Max]	94.0 [22.0, 224]	96.0 [36.0, 331]	94.5 [22.0, 331]
Missing	41 (4.0%)	7 (6.7%)	48 (4.2%)
**Triglycerides**			
Mean (SD)	146 (99.5)	148 (104)	147 (99.9)
Median [Min, Max]	120 [30.0, 913]	116 [40.0, 666]	120 [30.0, 913]
Missing	17 (1.7%)	3 (2.9%)	20 (1.8%)
**Insulin**			
Mean (SD)	19.0 (17.8)	20.1 (15.4)	19.1 (17.6)
Median [Min, Max]	13.4 [1.30, 162]	16.1 [2.20, 73.9]	13.5 [1.30, 162]
Missing	19 (1.8%)	4 (3.8%)	23 (2.0%)
